# What Is the Health and Well-Being Burden for Parents Living With a Child With ADHD in the United Kingdom?

**DOI:** 10.1177/1087054720925899

**Published:** 2020-06-19

**Authors:** Tessa Peasgood, Anupam Bhardwaj, John E. Brazier, Katie Biggs, David Coghill, David Daley, Cindy L. Cooper, Cyril De Silva, Val Harpin, Paul Hodgkins, Amulya Nadkarni, Juliana Setyawan, Edmund J. S. Sonuga-Barke

**Affiliations:** 1 School of Health and Related Research (ScHARR), Sheffield, UK; 2 Cambridgeshire and Peterborough NHS Foundation Trust, Cambridge, UK; 3 Clinical Trials Research Unit (CTRU), ScHARR, Sheffield, UK; 4 Department of Paediatrics & Department of Psychiatry, The University of Melbourne, Victoria, Australia; 5 Division of Psychiatry & Applied Psychology & Centre for ADHD and Neurodevelopmental Disorders Across the Lifespan, Institute of Mental Health, University of Nottingham, Nottingham, UK; 6 Medway NHS Foundation Trust, Kent, UK; 7 Sheffield Children’s NHS Foundation Trust, UK; 8 Global HEOR and Epidemiology, Wayne, PA, USA; 9 Lincolnshire Partnership NHS Foundation Trust, Lincoln, UK; 10 Arena Pharmaceuticals, San Diego CA, USA; 11 Institute of Psychiatry, Psychology and Neuroscience, King’s College London, London, UK

**Keywords:** ADHD, parents, carers, well-being, EQ-5D, Warwick Edinburgh Mental Well-Being Scale (WEMWBS)

## Abstract

**Objective:** To explore the burden associated with childhood ADHD in a large observational study. **Methods:** We recruited familes with at least one child (6-18 years) with ADHD via 15 NHS trusts in the UK, and collected data from all family members. We made careful adjustments to ensure a like-for-like comparison with two different control groups, and explored the impact of controlling for a positive parental/carer ADHD screen, employment, and relationship status. **Results:** We found significant negative impacts of childhood ADHD on parents’/carers’ hours and quality of sleep, satisfaction with leisure time, and health-related quality of life (measured by the EuroQol-5D [EQ-5D]). We found a decrement in life satisfaction, mental well-being (as measured by the Short–Warwick Edinburgh Mental Well-Being Scale [S-WEMWBS]), and satisfaction with intimate relationships, but this was not always robust across the different control groups. We did not find any decrement in satisfaction with health, self-reported health status, or satisfaction with income. **Conclusion:** The study quantifies the impact on the health and well-being of parents living with a child with ADHD using a survey of families attending ADHD clinics in the United Kingdom.

## Introduction

The main aim of this study was to identify any differences in health and well-being outcomes between parents/carers of a child with ADHD receiving usual treatment and carefully matched control parents/carers without a child with ADHD. This study also addressed two related questions. First, is the burden on health and well-being independent of the potentially confounding impact of parents’ own ADHD symptoms? Second, does parenting a child with ADHD differentially impact on different measures of health and well-being? By collecting information on a number of different well-being outcomes (overall life satisfaction, satisfaction with aspects of life and relationships, positive mental well-being) and health outcomes (health-related quality of life [EQ-5D] and sleep [which is also associated with well-being [Steptoe et al., 2008]]), we were able to develop an impact profile across multiple domains of well-being and health.

ADHD is a common childhood onset neurodevelopmental disorder, characterized by age-inappropriate levels of inattention and/or hyperactivity and impulsivity. A recent systematic review of prevalence rates, using the *Diagnostic and Statistical Manual of Mental Disorders* (4th ed.; *DSM-IV*; American Psychiatric Association, 1994) diagnostic criteria, gave estimates of between 5.9% and 7.1% of children worldwide; males were more likely than females to meet the criteria for a diagnosis of ADHD (Willcutt, 2012). Estimates of prevalence in the United Kingdom have tended to be slightly lower (Daley, 2006). ADHD symptoms often continue into adulthood with about two thirds of children having significant difficulties in adulthood (Faraone et al., 2006). ADHD has detrimental impacts upon the health, well-being, and education outcomes of children (Shaw et al., 2012). The additional stress of caring for a child with ADHD also has consequences for parents/carers. Parents who have children with ADHD have been found to experience lower mental health (Mash & Johnston, 1990), greater parenting-related stress (Baker & McCal, 1995), lower parenting self-esteem (Mash & Johnston, 1983), be more at risk of depression (Brown & Pacini, 1989), have increased levels of marital discord (Brown & Pacini, 1989), increased rates of divorce (Wymbs et al., 2008), and higher alcohol consumption (Pelham & Lang, 1999) than parents who do not have children with ADHD.

There are some important gaps in the literature regarding the impact of caring for a child with ADHD that this study aims to address. A key weakness of previous work has been a failure to adequately account for the clustering of ADHD in families. Parents of children with ADHD are more likely to have (or have had in the past) ADHD themselves (Faraone & Doyle, 2001), which may or may not have been diagnosed and treated. Adult ADHD symptoms have been associated with poorer satisfaction with life (Gudjonsson et al., 2009), anxiety and depression (Chao et al., 2008), poor sleep quality (Adler et al., 2009), and teenage pregnancy (Wehmeier et al., 2010). The lower well-being and health of parents with a child with ADHD may arise in part because of the parent’s own ADHD symptoms (or their spouse’s ADHD symptoms) rather than as a consequence of living with a child with ADHD. This study seeks to address this through the collection of a self-report measure of parent ADHD symptoms.

A further weakness in existing work has been the inadequate control for differences in personal and environmental factors. These vary between households and between those families with and without a child with ADHD. Families with one or more children with ADHD commonly face multiple adversities (Deault, 2010), such as lone parenthood and low maternal education (Hjern et al., 2010). Many of these negative environmental factors are thought to interact with genetic vulnerability to increase the risk of children developing ADHD (Thapar et al., 2012) and of developing subsequent comorbidities (Deault, 2010). This study addresses some of the complexities relating to the differences in environmental factors between families by controlling for exogenous characteristics (those that have not been caused by the presence of the child with ADHD, such as parental education) and considering the impact of potentially endogenous characteristics (those that might, at least in part, have been caused by the presence of the child with ADHD, such as parental relationship status).

For policymakers there is increasing interest in the impact of conditions and interventions on health-related quality of life using generic measures such as the EuroQol-5D (EQ-5D; EuroQol Group, 1990). These measures provide information that is required to determine the cost-effectiveness of new interventions in terms of cost per Quality Adjusted Life Year (QALY) by agencies such as NICE in the United Kingdom (National Institute for Health and Care Excellence, 2013) and related organizations in Australia, Canada, the Netherlands, and others (O’Donnell et al., 2009). There is little evidence within the existing literature on the impact of parenting children with ADHD on these measures. In recent years, there has been interest in the impact of health and health care on subjective well-being (Organization for Economic Co-Operation and Development, 2013), which has also been neglected in the ADHD literature.

Understanding and quantifying the effect of having a child with ADHD on parent/carer well-being is vital to clinicians providing services and to policymakers. Support to reduce any negative impact on well-being could reduce the need for additional health and social care input for families, increase the ability of carers to work and improve their resilience in caring for their child/children.

## Method

The study obtained ethical approval from the Sheffield NHS Research Ethics Committee, research governance was approved in each research site, and written consent was obtained from all participants.

## Participants

### ADHD-Family Group

Families were recruited from 15 centers (NHS trusts), which included routine clinics in Scotland and England (Coventry, Derby, Dundee, Durham, Leicester, Lincoln, Medway, Newcastle, Tyne area, North Essex, Nottingham, Rotherham, Sheffield, Southampton, South Staffordshire) between December 2010 and September 2012. Families were invited to participate in the study as a part of the ADHD-family group if they had a child (or children) aged 6 to 18 years, with a current diagnosis of ADHD and attending one of the centers/clinics. This sample aimed to be representative of a typical U.K. ADHD-clinic population. The sampling frame covered a wide geographical area and included both specialist child and adolescent mental health services (CAMHS) and pediatric clinics. The children had all received a clinical diagnosis of ADHD though centers and clinics varied in their diagnostic protocols. Children with a formal diagnosis of Conduct Disorder were excluded to maintain a tight focus within the study on ADHD. Conduct Disorder with ADHD may be etiologically distinct from ADHD alone (Faraone et al., 1997).

### Control Groups

To identify the burden imposed upon a family due to a child having ADHD, we ideally need to know what life would be like for that family had the child not developed ADHD. We cannot observe this counterfactual life, therefore the identification and recruitment of an appropriate control is essential. Various options have been used in the past to identify control groups for children with ADHD, such as requesting randomly identified individuals from the child’s school. However, this approach is unlikely to generate a control group which is sufficiently matched across the wide range of important household characteristics: parental age, education attainment, socio-economic status, employment status, and household composition. Our study used two different control groups.

#### Understanding Society (USoc) controls

The first control group was taken from Wave 1 (2009–2010) of the United Kingdom’s largest household longitudinal survey “Understanding Society,” a multi-topic survey conducted by the Institute for Social and Economic Research (ISER, University of Essex & National Center for Social Research, 2012). USoc is a nationally representative survey (Lynn et al., 2012), which provides a sample of more than 40,000 U.K. households to identify a control group that had completed some of the same measures used in the ADHD-family group. The control group was taken from the *general population* sample of adults aged more than 20 and below 70 years (1 year above and below the maximum and minimum age of ADHD-family group carers) living in a household in England or Scotland, with children aged from 6 to 18 years.^1^

#### South Yorkshire Cohort (SYC) controls

A second control group of families was recruited from the SYC^2^ (Relton et al., 2011), which recruited 18,000 patients (aged 16–85 years) via 40 general practitioner practices across South Yorkshire for a number of research projects from 2010. A sample of families from this cohort was invited via mail to participate in the study; those who responded positively were sent the full set of questionnaires by post. These families completed the same survey instruments as the ADHD-family group.

For both control groups, we used careful matching procedures (explained below) to ensure a balance in key characteristics. These characteristics included parental education, gender, and age, which were unlikely to have been impacted upon by the presence of a child with ADHD. There are strengths and weaknesses to each of the two control groups. The “Understanding Society” control group offers a very large sample from which to identify a large well-matched control group, but not all of the outcome measures (e.g., EQ-5D) are included in this survey, and there is no measure of either child or adult ADHD symptoms. The SYC control is a smaller sample, but it used all of the same health and well-being questionnaires including the adult ADHD rating scale and EQ-5D. The two control groups had different recruitment methods, and this is likely to have resulted in some differences between the samples. Because we rely on observational data, there is a risk of unobservable differences between our ADHD-family group and the control non-ADHD-family group (e.g., attitudes toward parenting) that could influence the findings. Using two different control groups provides us with an important robustness check that can reduce this risk.

## Instruments

### EQ-5D

The EQ-5D (EuroQol Group, 1990) is a self-report, generic preference-based quality of life instrument used to estimate health state utilities on a 0 to 1 scale, where 0 represents a health state as bad as being dead and 1 represents one as good as full health. This measure is used to calculate QALYs: a single measure combining quality of life and length of life for use in cost-effectiveness analysis of health technologies (National Institute for Health and Care Excellence, 2013). The EQ-5D instrument comprises five questions dealing with various aspects of physical and mental health (mobility, self-care, usual activities, pain/discomfort, anxiety/depression), for which the response to each is one of three degrees of impairment. Data from the EQ-5D questionnaire can be converted to an EQ-5D utility index using various scoring algorithms. Here, utility weights are derived from a valuation exercise conducted in the United Kingdom (Dolan, 1997). The EQ-VAS, usually completed alongside the EQ-5D, is visual analogue scale (VAS) for recording an individual’s rating for their current health. This is anchored at the bottom at 0 (*worst imaginable health state*) and at the top at 100 (*best imaginable health state*).

### Short–Warwick Edinburgh Mental Well-Being Scale (S-WEMWBS)

S-WEMWBS is a self-report, seven-item scale that measures positive mental well-being (Stewart-Brown et al., 2009). Questions are asked on the following: optimism, feeling useful, feeling relaxed, feeling able to deal with problems, thinking clearly, feeling close to others, and feeling decisive. Each item is scored between 1 (“*none of the time*”) and 5 (“*all of the time*”), giving a maximum total score of 35 (hence a high score indicates better mental well-being). There are no “cutoff” points in the scoring scale. A linear transformation of the raw score is recommended for parametric analysis (Stewart-Brown et al., 2009) and is applied here.

### Life Satisfaction

Respondents completed four questions about satisfaction with health, satisfaction with the amount of leisure time, satisfaction with income, and satisfaction with life overall. Each question uses a 1 (*completely dissatisfied*) to 7 (*completely satisfied*) scale.

### Sleep

Three questions were asked about the quality of carers’ sleep; how often during the last month they had trouble sleeping due to not being able to fall asleep within 30 min and due to waking during the night or early in the morning, each with five response categories: and a question on their overall perception of sleep quality, with four response categories (very good, fairly good, fairly bad, very bad). These responses were combined using factor analysis to create a sleep problems index. Respondents were also asked about their usual hours of sleep per night over the last month. These questions were taken from the U.K. survey “Understanding Society.” These were chosen because they had been subject to rigorous piloting (Boreham et al., 2012).

### Relationship With Partner

A question concerning the overall happiness with the carers’ relationship with their partner was taken from “Understanding Society.” This has seven responses (extremely unhappy, fairly unhappy, a little unhappy, happy, very happy, extremely happy, and perfect).

### ADHD: Adult Self Report Scale

To control for the effect of the adult having ADHD symptoms, the Adult Self Report Scale (ASRS v1.1; Kessler et al., 2005) was completed by parents/carers. This is a six-item screening tool, with five responses for each item. Where an individual has four or more positive responses, this is taken as indicating possible adult ADHD. This scale has been found to have a sensitivity of 1.0 and a specificity of 0.71 in a U.K. primary care setting (Hines et al., 2012). Among population of substance users, the specificity of the screen has been found to be much lower (Chiasson et al., 2011) and it is unclear what the specificity of the screen is for people with mental health problems, but it is known that depression and adult ADHD are often comorbid (Kessler et al., 2006). Parents/carers were also asked whether they were ever diagnosed with ADHD in the past.

### ADHD Rating Scale (IV)

Parents/carers completed the ADHD rating scale ^(^DuPaul et al., 1998^)^ which asks 18 questions about the child’s behavior over the last 6 months, each with four response categories. This generated a score from 0 to 54.^3^

## Statistical Analysis

Due to differences in background characteristics between the ADHD-family group and the control groups, we used a process called Coarsened Exact Matching (CEM; Blackwell et al., 2009; Iacus et al., 2012). This process generates closely matched comparisons which can improve subsequent regressions (Ho et al., 2007). Individuals were allocated to a subgroup based on gender, highest education attainment (degree or diploma, nursing or teaching qualification or equivalent/A level or equivalent/O level or GCSE or equivalent/no formal qualifications), and age (<40/40–59/60+ years): all characteristics that are not caused by caring for a child with ADHD. Individuals in the ADHD-family group and the control groups were only included in the analysis where a match was present in their subgroup. The control observations were then assigned a weight in proportion to the number of ADHD-family group and control group observations that are present within each subgroup, and normalized to ensure the sample size remains the same. All members of the ADHD-family group were assigned a weight of one. The matching process (pruning and weighting) creates a better covariate balance between the ADHD-family group and the control groups, and any remaining imbalance in observed variables is further controlled for using standard appropriate weighted regression models. The more accurate the match, the less burden is put on getting the assumptions implicit in the regression models correct; hence, it is less sensitive to choices about whether to include interaction or higher order terms, for example. Due to discarding data that do not have a good match, the model is not extrapolating counterfactual outcomes to areas where we do not have good information. Throughout the matching and the regression adjustment, we still rely on an assumption that there are no important “unobservable” differences between the families with a child with ADHD and those without.

The study collected a broad range of individual carer characteristics (age, gender, the number of children in the household, education level, and employment and income deprivation within the local area) to control for important influences on health and well-being. In particular, comparisons to the SYC control were estimated with and without controlling for the adult ADHD screen to explore the impact of possible parental ADHD on the burden generated by the child with ADHD. The USoc-control group did not contain information on adult ADHD; hence, we were not able to include the adult ADHD screen as a control. This may result in an overestimation of the burden on parents. We addressed this potential bias by making two comparisons with the USoc data, one with the full sample and one with a smaller subsample that excluded those adults in our ADHD-family group with a positive ADHD screen or who had previously been diagnosed with ADHD.

We also estimated models with and without other variables that may be affected by the presence of a child with ADHD. Relationship and employment status have a substantial impact on health and well-being outcomes, but these could be caused in part by living with a child with ADHD. Those parents/carers who are most impacted by caring for a child with ADHD are likely also to be those parents who are unable to maintain employment. Kvist et al. (2013) identified a considerable impact on parental labor outcomes and relationship dissolution from caring for a child with ADHD in Denmark. Therefore, controlling for these family level factors may produce an underestimate of the full impact of living with a child with ADHD. If a parent’s failed relationship is in part caused by having a child with ADHD then at least some of the detrimental impact of relationship status upon health and well-being can be attributed to the presence of the child. There may be a similar issue arising from controlling for the adult ADHD screen because ADHD symptoms could be exacerbated by having a child with ADHD. The true impact of having a child with ADHD is likely to lie between the impact found with the employment, relationship status, and adult ADHD screen controls and the impact found without those controls. We anticipated that the impact of the child with ADHD on an outcome may differ between primary and secondary carers, and therefore included an interaction term for being a secondary carer and having a child with ADHD in addition to a dummy variable for being a secondary carer.

Each parent outcome measure was treated as a dependent variable and modeled as a function of individual and family characteristics and the presence of a child with ADHD. We adopted methods suitable to each outcome measure in question, with consideration given to the nature and distribution of the measure. The S-WEMWBS and the EQ-VAS reported hours sleep, and the life and domain satisfactions were analyzed using linear models (ordinary least squares [OLS]), the EQ-5D was analyzed using a nonlinear tobit model due to the bounded nature of the scale (it cannot go above 1) and the substantial proportion of responses being at one (Kvist et al., 2013). Whether the parent/carer was in a relationship was considered using a logit model.

The sleep problems index was created using factor analysis. Using the Kaiser criterion of retaining factors with an Eigen value of 1 or more, a single factor was retained (at least 69% of the variance was explained by this factor). Factor loadings were used to create a linear composite variable (all loadings were above 0.80). The index had an acceptable internal consistency with Cronbach’s alphas of above .74. The sleep problems index was analyzed using OLS.

Given the multiple testing and large sample size, we rely upon a 1% significance level.

## Results

### Study Population

In total, 549 families with a child with ADHD consented to the study, but despite having given consent, no self-completed were obtained for 94 families, leaving 455 families with 635 parents/carers (one carer was excluded as he was the boyfriend of the individual with ADHD). Of these, 428 were primary carers and 208 were secondary carers. Data were missing for 31 parents/carers, leaving a final sample size in the ADHD-family group of 604. In total, 10,718 carers were identified from USoc, of which 2,123 had either no outcome data or were missing key data on covariates, leaving 8,595 eligible participants. In total, 123 families from the SYC were recruited, from which we have data on 227 parent/carers (123 were primary carers), of which 12 had missing data, leaving 215 participants. The children diagnosed with ADHD of the parents/carers included in this analysis were mostly boys (83%), aged on average 11.8 years (range 6–18 years), with an average parent completed ADHD rating scale score of 41.2 (*SD* = 10.6). Background details on the samples prior to the matching are described in Table 1 (further descriptive data on the outcome measures can be found in the online supplement Table S1). The SYC-control group contained parents/carers who were slightly older, more likely to be male, with greater education attainment, fewer single parents, with fewer children living in household and more likely to be employed. The USoc-control group shared more similar characteristics to the ADHD-family group, although differences in employment were still present.

**Table 1.** Background Characteristics of Parents/Carers (Prior to Matching).

Individual carer variables	ADHD group *N* = 604 (unless otherwise specified)	South Yorkshire Cohort (SYC) *N* = 215 (unless otherwise specified)	Understanding Society (USoc) *N* = 8,595
Age (*M*) years	41.4 (*SD* 7.9) range 22 to 68	44.6 (*SD* 6.0) range 25 to 62	41.2 (*SD* 7.9) range 21 to 69
Male	200 (33.1%)	96 (44.7%)	2,967 (34.5%)
Primary carer	407 (67.4%)	122 (56.7%)	5,776 (67.2%)
No formal qualifications	104 (17.2%)	5 (2.3%)	1,522 (17.1%)
Up to O level	269 (44.5%)	41 (19.5%)	3,283 (38.2%)
A level or equivalent	67 (11.1%)	29 (13.5%)	685 (8.0%)
Further or higher education	164 (27.2%)	139 (64.7%)	3,105(36.1%)
Do not have a job	288 (47.7%; *N* = 601)	22 (10.2%)	2,529 (29.7%)
Meet cutoff for adult ADHD	33.3% (*N* = 574)	7.4% (*N* = 196)	Not available
Past diagnosis of ADHD	3% (*N* = 600)	0% (*N* = 215)	Not available
Household variables	*N* = 407	*N* = 122	*N* = 5,776
Local area employment deprivation^a^	10.6 (*SD* 6.5)	8.3 (*SD* 6.4)	10.0 (*SD* 6.7)
Local area income deprivation^a^	17.0 (*SD* 11.1)	11.6 (*SD* 10.6)	16.9 (*SD* 12.5)
Number of children in the household	2.3 (*SD* 1.1) range 1 to 9	1.8 (*SD* 0.7) range 1 to 4	2.0 (*SD* 0.99) range 1 to 12
Number of children with ADHD	1 child: 352 2 children: 45 3 to 5 children: 10	None	Not known
Number of single parent/carer households	132 (32.5%) *N* = 406	18 (14.5%)	1,588 (27.9%)

a Local area employment deprivation is the 2010 proportion of working age population in the Lower Level Super Outcome Area (LSOA) in England and at the slightly smaller data zone level for Scotland. The local area income deprivation was derived from the 2010 proportion of the population income deprived according to benefit claims at the LSOA in England and the data zone for Scotland.

These differences in initial carer characteristics (before the data are matched) highlight the importance of the matching procedure and the need to include covariates within the regression models. The matching process resulted in dropping 89 of the 604 ADHD-family group carers when making the comparison to the SYC-control group (with 1 pruned from the SYC). Most of this arises because of the lack of adequate matches within the SYC-control group for primary carers without any formal education qualifications. For the USoc comparison, none of the ADHD-family group carers were pruned and only 35 out of 8,595 USoc cases were pruned.

### Impact of the Child With ADHD

The matched and adjusted comparisons of ADHD-family group carers to the USoc-control group for each outcome measure are shown in Table 2 (Columns 1–4). Column 1 shows the marginal effect of living with a child with ADHD using the matched, weighted sample and additionally controlling for the standard covariates of parental age, parental education, income, and employment deprivation in the area and number of children in the household. A dummy for being a secondary carer and an interaction effect for being a secondary carer and having a child with ADHD are also included. Column 2 additionally controls for employment and relationship status. As the USoc dataset does not contain information on adult ADHD symptoms, an additional comparison was run which excluded those parents/carers in the ADHD-family group who report a past diagnosis of ADHD, or reach a cutoff for possible ADHD according to the adult ADHD screening tool. This resulted in 209 carers being excluded. These findings are reported in Columns 3 and 4.

**Table 2.** Health and Well-Being Impacts (Marginal Effects) of Living With a Child With ADHD on Health and Well-Being Using Matched and Weighted Regressions.

Variables	Understanding society control group				South Yorkshire Cohort control group		
	Full sample		Subsample robustness check. Only carers in the ADHD-family group without suspected ADHD				
	(1) Standard covariates	(2) Standard covariates plus job and relationship status	(3) Standard covariates	(4) Standard covariates plus job and relationship status	(5) Standard covariates	(6) Standard covariates plus ADHD screen	(7) Standard covariates plus ADHD screen, job and relationship status
EQ-5D							
Child with ADHD					−0.071***	−0.050**	−0.018
Secondary carer					0.005	0.013	−0.007
Adult ADHD possible						−0.067***	−0.057***
Partner at home							0.033
No job							−0.129***
*N*					724	685	682
EQ-VAS							
Child with ADHD					−3.856	−0.837	2.900
Secondary carer & child with ADHD					3.214	5.816	8.334*
Secondary carer					3.341	3.291	0.500
Adult ADHD possible						−6.433***	−5.595***
Partner at home							3.662
No job							−12.244***
*N*					713	678	675
Adjusted *R*^2^					.056	.075	.152
S-WEMWBS							
Child with ADHD	−1.573***	−1.362***	−1.192***	−0.973***	−1.457*	−0.927	−0.398
Secondary carer & child with ADHD	0.398	0.253	0.761**	0.591	0.237	0.787	1.168
Secondary carer	0.518*	0.356	0.477*	0.323	0.071	0.027	−0.407
Adult ADHD possible	—	—	—	—		−1.335***	−1.225***
Partner at home		0.743***		0.756***			0.724
No job		−0.891***		−0.938***			−1.608***
*N*	8,830	8,670	8,626	8,467	715	680	677
Adjusted *R*^2^	.033	.048	.026	.042	.100	.127	.164
Life satisfaction							
Child with ADHD	−0.405***	−0.327***	−0.179**	−0.111	−0.581**	−0.398	−0.154
Secondary carer & child with ADHD	0.165	0.119	0.170	0.126	0.059	0.283	0.462
Secondary carer	0.321***	0.198*	0.295**	0.179	0.325	0.291	0.012
Adult ADHD possible	—	—	—	—		−0.541***	−0.457***
Partner at home		0.418***		0.421***			0.739***
No job		−0.384***		−0.400***			−0.522***
*N*	9,015	8,845	8,807	8,638	725	687	684
Adjusted *R*^2^	.068	.087	.031	.057	.055	.082	.143
Health satisfaction							
Child with ADHD	−0.260***	−0.132	−0.067	0.075	−0.171	0.114	0.384
Secondary carer & child with ADHD	0.055	−0.020	0.137	0.032	0.439	0.602*	0.788**
Secondary carer	0.035	−0.001	0.016	−0.015	0.069	0.107	−0.125
Adult ADHD possible	—	—	—	—		−0.589***	−0.539***
Partner at home		0.137**		0.159***			0.306
No job		−0.630***		−0.642***			−0.826***
*N*	9,016	8,844	8,808	8,637	726	688	685
Adjusted *R*^2^	.027	.054	.025	.053	.044	.071	.126
Income satisfaction							
Child with ADHD	0.015	0.128	0.226**	0.315***	−0.332	−0.140	0.115
Secondary carer & child with ADHD	−0.024	−0.098	−0.014	−0.077	0.274	0.570*	0.736**
Secondary carer	0.332***	0.187	0.316***	0.169	−0.104	−0.169	−0.364
Adult ADHD possible	—	—	—	—		−0.538***	−0.458***
Partner at home		0.604***		0.638***			0.540***
No job		−0.533***		−0.535***			−0.676***
*N*	9,006	8,838	8,799	8,632	726	688	685
Adjusted *R*^2^	.059	.097	.062	.102	.059	.081	.129
Leisure satisfaction							
Child with ADHD	−0.784***	−0.809***	−0.470***	−0.501***	−1.087***	−0.936***	−0.902***
Secondary carer & child with ADHD	0.412***	0.425***	0.334**	0.356**	−0.429	−0.202	−0.171
Secondary carer	0.139	0.088	0.148	0.100	0.235	0.178	0.103
Adult ADHD possible	—	—	—	—		−0.480***	−0.463***
Partner at home		0.189***		0.186***			0.326**
No job		0.109**		0.125**			0.016
*N*	9,014	8,845	8,806	8,638	726	688	685
Adjusted *R*^2^	.028	.031	.019	.022	.116	.130	.136
Hours sleep							
Child with ADHD	−0.804***	−0.768***	−0.665***	−0.642***	−0.517***	−0.407**	−0.224
Secondary carer & child with ADHD	0.280**	0.269**	0.278*	0.274*	0.223	0.230	0.364
Secondary carer	0.002	−0.057	0.017	−0.036	−0.177	−0.147	−0.277
Adult ADHD possible						−0.325**	−0.264**
Partner at home		0.183***		0.173***			0.400**
No job		−0.218***		−0.202***			−0.361**
*N*	8,680	8,492	8,483	8,296	700	662	659
Adjusted *R*^2^	.033	.042	.022	.030	.075	.085	.111
Sleep problem index							
Child with ADHD	0.720***	0.652***	0.577***	0.514***	0.389**	0.314*	0.167
Secondary carer & child with ADHD	−0.115	−0.089	−0.086	−0.061	−0.047	−0.082	−0.192
Secondary carer	−0.045	0.018	−0.034	0.019	0.014	−0.008	0.100
Adult ADHD possible	—	—	—	—		0.348***	0.312***
Partner at home		−0.165***		−0.160***			−0.206
No job		0.340***		0.352***			0.448***
*N*	8,280	8,106	8,110	7,936	647	612	609
Adjusted *R*^2^	.0597	.0857	.0453	.0717	.123	.151	.197
In a relationship? (primary carers only)							
ADHD child	−0.056**	−0.036	−0.010	0.010	−0.142**	−0.146**	−0.115
Adult ADHD possible	—	—	—	—		−0.137***	−0.130***
No job		−0.094***		−0.095***			−0.110*
*N*	6,091	6,040	5,924	5,876	467	454	451
Relationship happiness (those in a relationship only)							
Child with ADHD	−0.322***	−0.289***	−0.244***	−0.211**	−0.360**	−0.390**	−0.322*
Secondary carer^a^	0.051	0.056	−0.047	−0.039	0.037	−0.014	−0.037
Adult ADHD possible	—	—	—	—		−0.056	−0.039
Partners ADHD possible	—	—	—	—		−0.123	−0.072
No job		−0.177***		−0.148***			−0.309*
*N*	7,225	7,150	7,067	6,992	595	465	464
Adjusted *R*^2^	.0198	.0222	.0185	.0203	.0408	.0377	.0418

*Note.* The EQ-5D model uses a weighted tobit and shows average marginal effects which have incorporated interaction effects. The cohabiting relationship model uses logit and again shows average marginal effects. The other models use weighted OLS. Controls include gender, age, number of children in the household, highest education attained (further degree / first degree or equivalent /A level or equivalent / O level or equivalent / base category: no formal qualifications), percent employment deprived in the area, and percent income deprived. A constant is also included. The relationship happiness regression is based on a separate matching run, which does not include those without a partner at home. Full details of these regressions are available from the authors. EQ-VAS = EuroQol visual analogue scale; S-WEMWBS = Short–Warwick Edinburgh Mental Well-Being Scale; OLS = Ordinary least squares.

a Based on Akaike Information Criterion (AIC), the model performed better without the interaction term.

* *p* < .1. **p < .05. ***p < .01, these are based on robust standard errors which are clustered at the household level.

The USoc comparison found that carers of a child with ADHD had S-WEMWBS scores that were 1.57 points lower on the 7 to 35 scale (about 0.4 of a *SD*). Controlling for employment and relationship status reduced the effect to 1.36 lower and using the non-ADHD screen subsample reduced these effect sizes to 1.19 and 0.97, respectively. Additional analysis (described in the online supplement Table S2) looking at each of the item responses found that the ADHD-family group carers report less favorable outcomes on all S-WEMWBS questions.

In the full sample comparison, *life satisfaction* was significantly lower (–0.41, on a 1–7 scale, about 0.3 of *SD*) for the ADHD-family group. This reduced to –0.33 when the relationship and employment controls were added. When carers who screened positive for ADHD were removed, the effect was reduced to –0.18 and non-significant when employment and relationship status were included with this subsample (Column 4). The ADHD-family group reported lower *health satisfaction* (–0.26, on a 1–7 scale); however, this effect was not robust to the inclusion of employment and relationship status, nor in the smaller negative-ADHD screen subsample. No differences were identified for *income satisfaction* in the full sample, but in the non-ADHD screen subsample, the ADHD-family group had higher levels of income satisfaction compared with the matched controls. The ADHD-family group, particularly primary carers, report significantly lower *satisfaction with leisure time* (0.47–0.81 lower depending upon model, on a scale of 1–7). This leisure time effect was robust to the inclusion of the additional controls.

Carers in the ADHD-family group also reported less sleep (39–48 min a night for primary carers and 22–31 min for secondary carers) than those in the USoc-control group, again with the additional controls and non-ADHD screen subsample showing a slightly reduced effect. They also had a higher sleep problems index: from 0.51 to 0.72 of a standard deviation higher, depending on the model.

There were significantly more single parent/carers in the ADHD-family group than in the USoc-control group, but this difference was not robust to controlling for employment status, nor apparent in the non-ADHD screen subsample. Those carers in the ADHD-family group that were in a relationship were significantly less happy with their relationship than carers in the USoc-control group: 0.32 lower (on a 1–7 scale) reducing to 0.29 lower when controlling for employment status and 0.24 lower when using the subsample that excludes those with a positive adult ADHD screen.

The matched and adjusted comparisons of the ADHD-family group to the SYC-control group are also shown in Table 2 (Columns 5–7). Column 5 shows the marginal effect of having a child with ADHD while controlling only for the set of standard covariates; Column 6 includes the carers’ ADHD symptom screen as an additional control; and Column 7 includes controls for employment and relationship status.

We found a significant negative impact (–0.071) of the presence of a child with ADHD on the EQ-5D (which is scored such that 1.0 is equivalent to 1 year spent in good health, 0 is equivalent to being dead). This decreased by approximately one third following the inclusion of the carers own ADHD screen (–0.050) and was nonsignificant when the relationship and employment controls were included. Additional analysis on the individual items of the EQ-5D found that this effect was driven by differences in the self-care item and the anxiety and depression item (see Table S2 in the online appendix). The EQ-VAS health measure was unaffected by having a child with ADHD.

The comparison to the SYC-group matched controls found that the S-WEMWBS was not significantly lower for parents/carers of a child with ADHD. Considering the individual questions within the S-WEMWBS (see Table S3 in the online appendix), the ADHD-family group carers reported being significantly less relaxed, less optimistic, and less able to deal with their problems, but no differences were identified for the other four items.

The ADHD-family group reported lower (at 5% significance) *life satisfaction* (–0.58 on the 1–7 scale), but this was not robust to the inclusion of the adult ADHD screen. They reported similar *health and income satisfaction* to matched controls in the SYC, with secondary carers with a child with ADHD expressing higher satisfaction with their health and income. In line with the USoc comparison, ADHD-family carers also reported substantially lower *leisure satisfaction* (–0.94 when adult ADHD is controlled for).

The ADHD-family group reported fewer hours sleep than the SYC-group. This effects remains even after controlling for the carers own ADHD screen (about 25 min less per night), though this is no longer significant once the relationship and employment controls are included. Carers with a child with ADHD reported a higher sleep problems index, though this was not robust to the inclusion of the adult ADHD screen. Interestingly, carers without employment or without a cohabiting partner have notably higher problems with their sleep. Nearly half of the ADHD-family group primary carers are woken during the night by their child with ADHD, with more than 10% being woken three or more times (data are available in the online appendix Table S1).

The ADHD-family group had more single parent/carers than the SYC control when the data are matched though this effect was not robust to the inclusion of employment status. Carers of a child with ADHD who were in a conjugal relationship were less happy with their relationship (–0.36 lower on a 1–7 scale). This difference was robust to the inclusion of the adult ADHD screen and their partner’s ADHD screen, but was only significant at the 10% level once controlling for employment status.

Having a positive adult ADHD screen had a significantly detrimental impact upon all outcome measures. Not having employment also showed a significant detrimental impact upon all outcome measures with the exception of leisure satisfaction and the probability of being in a relationship.

## Discussion

Our key findings are summarized in Table 3 which shows where a significant decrement of caring for a child with ADHD was identified across the different measures (*p* < .01, unless shown as *p* < .05). When using the USoc-control group, we found that having a child with ADHD reduced parent/carer overall satisfaction with life and mental well-being. We also identified a negative impact on satisfaction with leisure time and happiness with relationships, sleep hours, and sleep quality. These effects tended to be weaker using the SYC as a control which may be a consequence of its smaller sample size. When comparing the standard models without the adult ADHD screen, the magnitude of the deficit for carers with a child with ADHD is broadly similar between the two control group comparisons (e.g., life satisfaction –0.41 vs. –0.58; S-WEMWBS –1.57 vs. –1.46).

**Table 3.** Summary of Key Findings.

Variables	USoc				SYC		
	Full sample		Smaller sample excluding suspected adult ADHD in the ADHD-family group		Main controls	Plus adult ADHD screen	Plus adult ADHD screen & job and relationship
	Main controls	Plus job & relationship	Main controls	Plus job & relationship			
EQ-5D	NA	NA	NA	NA	√	√ (5%)	x
EQ-VAS	NA	NA	NA	NA	x	x	x
S-WEMWBS	√	√	√	√	x	x	x
Life satisfaction	√	√	√ (5%)	X	√ (5%)	x	x
Health	√	x	x	X	x	x	x
Income	X	x	Opposite	Opposite	x	x	x
Leisure	√	√	√	√	√	√	√
Hours sleep	√	√	√	√	√	√ (5%)	x
Sleep problem index	√	√	√	√	√ (5%)	x	x
In a relationship	√ (5%)	x	x	X	√ (5%)	√ (5%)	x
Relationship happiness	√	√	√	√ (5%)	√ (5%)	√ (5%)	x

*Note.* USoc = Understanding Society; SYC = South Yorkshire Cohort; EQ-VAS = EuroQol visual analogue scale; S-WEMWBS = Short–Warwick Edinburgh Mental Well-Being Scale.

Our findings reinforce the impact of caring for a child with ADHD on parental health as measured by the EQ-5D mental well-being and life satisfaction but no significant impact was identified upon health satisfaction or on the EQ-VAS which was contrary to our expectations. Interestingly, we identified a large and significant deficit in the EQ-5D (–0.071), which reduced slightly (–0.050) but remained significant at 5% following the inclusion of the adult ADHD screen. This magnitude is substantial relative to the minimally important difference (MID) for the EQ-5D (Cameron & Trivedi, 2009). This is despite the fact that the dimensions of the EQ-5D may not be the most relevant to carer-related quality of life. For example, the EQ-5D does not include relationship issues that are part of the CarerQol measure (Walters & Brazier, 2005), or activities that are part of the Carers Experience Scale (Brouwer et al., 2006). The decrement in the EQ-5D was driven by differences in anxiety/depression and self-care items (washing or dressing). A difference in self-care arising as a consequence of the caring role is a little surprising. Furthermore, if there was a difference in physical capability for self-care between the groups, one may expect this to also show up in differences in the EQ-VAS and health satisfaction, yet we found no difference in these variables. One possibility is that this refers to the time carers have available for washing/dressing; in which case the utility tariff, which is based on having a physical or mental health state which makes these tasks difficult, may not be appropriate. Consequently, the difference in the EQ-5D should be treated with caution.

The ADHD-family group carers reported fewer hours of sleep in both comparisons which is not surprising given the sleep problems experienced by many children with ADHD (Al-Janabi et al., 2011). In the USoc comparison, the loss of sleep is significantly greater for primary carers compared with secondary carers, but this pattern is not replicated in the SYC comparison. The high frequency of being woken during the night by the child with ADHD suggests they are likely to be at least partly responsible for this sleep deficit. There may, however, be other unobservable factors that influence sleep across the family, such as differences in coherence to routines or differences in screen time. The adult ADHD screen, primary carer relationship, and employment status all significantly impact upon both hours of sleep and the index of sleep problems. Furthermore, their inclusion within the models alters the magnitude of the effect of caring for a child with ADHD on sleep outcomes. This suggests that to fully understand the impact of caring for a child with ADHD on sleep behavior requires a whole family approach, giving consideration to broader family circumstances.

We find some indication of less happy intimate relationships and more single parents within the ADHD-family group, but given the potential for reverse causality here (poor parental relationships and relationship breakdown may be a risk factor in the child developing ADHD), this link is best established through a future analysis of longitudinal data.

All models found parents/carers of a child with ADHD had lower *satisfaction with leisure time*. This negative impact on leisure time is borne out by clinical experience. Many families report being reluctant to go out for a meal or a daytrip to amusement parks, for example, due to difficult experiences in the past. Children with ADHD find it very difficult to manage their behavior in these unstructured situations.

No negative impact was identified on *income satisfaction*. Indeed, the ADHD-family group reported higher income satisfaction than the USoc controls. We do not have a good explanation for this, although it could arise from favorable comparisons the ADHD-family group made to other domains of their life. In their longitudinal analysis of the United Kingdom’s Millennium Cohort study, Russell et al. (2014) found that income did not decrease for parents of children with ADHD (without comorbidities) compared with controls over the 7-year study period.

The USoc comparison finds greater impact in sleep and leisure satisfaction for primary carers than secondary carers. No obvious pattern was detected in the SYC comparison—which may, in part, result from selection effects into the study, with those secondary carers most impacted by the ADHD being those more likely to participate in the study.

The inclusion of the adult ADHD screen as a control in the SYC comparison did not impact on findings on sleep or leisure satisfaction but did lead to a slightly reduced effect size for life satisfaction, EQ-5D, and mental well-being. With regard to the USoc comparison, excluding those with a positive ADHD screen resulted in a loss of magnitude in differences in life satisfaction, mental well-being, satisfaction with leisure time, sleep problems, and relationship happiness. This suggests that some of the burden in these areas is being driven by symptoms of ADHD experienced by the adult carer. The adult ADHD screen was negatively related to all of the outcome measures. This is strongly indicative of adult ADHD having a detrimental impact upon health and well-being. However, the inclusion of the adult ADHD screen in this study was to act as a control variable. To get a clearer idea of the magnitude of the health and well-being burden of adult ADHD would require clinical confirmation of the presence of adult ADHD.

### Strengths

This study has a number of advantages over the existing literature. Foremost is the size of the study sample and the comparison to two separate control groups. The size of the control groups, particularly the USoc data, means we can afford to prune cases where a comparison would not be based on like for like. The control groups had extensive background details on the families, including socioeconomic information. The weighting of cases within the matching and additional covariate further controls for observed differences between the ADHD-family group and our controls. We have shown that prior to the matching, observed characteristics differ among the groups, and how the inclusion of the adult ADHD screen can alter findings, suggesting the potential for bias within past research which does not adequately control for these covariates.

The study used a number of different outcome measures, including those which have particular policy relevance such as the EQ-5D, not previously reported. The inclusiveness of outcome measures and adequate adjustments provide a more comprehensive and accurate estimation of the main impacts of having a child with ADHD experienced by parents/carers.

The advantages of both large sample size and using a number of different outcome measures simultaneously raise the risk of identifying spurious significant findings. We address this through focusing on a 1% significance level and looking for consistent findings across different control groups and covariate adjustments while cautious against over-reliance on any single significant finding.

### Limitations

The ADHD-family group contains those who have children that have been diagnosed with ADHD, are currently receiving some intervention, consent to being in the study and complete the required surveys; hence, they may not adequately represent all families in the United Kingdom with a child with ADHD. The dropout rate from consent to filling in the questionnaires was fairly high (17%), and this may be related to the extent of difficulties the families are currently experiencing. The completion rate of those who returned questionnaires was good (95% of carers had sufficient data to be included in the EQ-5D analysis): in part due to home visits by research nurses.

Our analysis relies on the assumption that children in the control groups did not have ADHD (diagnosed or undiagnosed). For the SYC-control group, the self-reported presence of a child with ADHD was an exclusion criteria. The USoc-control group was a representative sample of parents/carers in the United Kingdom, hence potentially includes families with ADHD children. This would lead to an underestimate of the impact of having a child with ADHD, but because the prevalence for childhood ADHD is likely to be around 4% (Daley, 2006), this should not have any great impact upon the results. We excluded patients with a co-diagnosis of Conduct Disorder; however, it is possible that other children in the sample had Conduct Disorder but had not been formally diagnosed.

This analysis was based on observational, cross-sectional data and therefore cannot be used to imply causality. Although the control groups were closely matched to the ADHD-family group in terms of observable characteristics and further model adjustments were implemented, we cannot be certain that there are not differences in unobserved characteristics which have not been accounted for. A potential risk factor in developing ADHD is the presence of toxins while in the womb arising from maternal smoking (Russell et al., 2014) or alcohol consumption (Button et al., 2005) during pregnancy (Mick et al., 2002). These behaviors have a strong relationship with health and well-being and are unobserved variables within our data. It could be that mothers of a child with ADHD have a higher prevalence of these behaviors and/or particular attitudes toward health that contribute to their lower health and well-being independent of the consequences of living with a child with ADHD. Because health behaviors are correlated between spouses, this could apply to both men and women. Negative parenting style has also been linked to the development of ADHD in children (Deault, 2010). If this style of parenting is more common among parents with poorer initial well-being, this could be confounding our results. There may also be unobservable differences in the way in which the questionnaires were completed. The study questionnaire unavoidably focuses the ADHD-family respondent on the consequences of living with a child with ADHD. Furthermore, some families may have perceived an incentive to overstate their problems. However, this incentive and framing is present across all questions, and yet we only identified an effect on some of the outcome measures, suggesting more targeted and thoughtful responses.

In the SYC-control comparison, we control for adult ADHD using the ASRS screening scale cutoff, and for the USoc comparison, we consider a subsample comparison of those carers who do not reach the cutoff on the ASRS screen and have not been diagnosed with ADHD in the past. However, in both cases, this does not account for adults who have experienced (undiagnosed) ADHD symptoms in the past but do not currently do so. Undiagnosed ADHD symptoms in the individual’s childhood/adolescence, even if no longer present, may have had a scarring impact upon health, education, employment, and well-being outcomes. If past ADHD has had a scarring impact upon health, and is not adequately controlled by the ASRS screen, this may result in an overestimate of the burden of caring for a child with ADHD. On the other hand, having a child with ADHD (or associated lack of sleep) may exacerbate adult ADHD symptoms in which case controlling for adult ADHD symptoms may underestimate the burden. Including a dichotomous control for possible adult ADHD may also risk controlling for something other than adult ADHD. Full diagnosis of adult ADHD requires a subsequent clinical interview of those individuals with a positive ASRS screen, and the fairly low specificity of the screen (0.71 in U.K. primary care [Hines et al., 2012]) could mean some false positives. Hence, we may have controlled for the presence of ADHD *or* the presence of a different problem (such as mental health problems, or parental stress). If this was the case then both the covariate dummy in the SYC regressions and the exclusion of individuals in the USoc comparison could possibly be over controlling and result in an underestimate of the true effects. While the adult ADHD control helps us understand the impact of caring for a child with ADHD, it is, nevertheless, the actual health and well-being of parents/carers of a child with ADHD that is most relevant for clinical practice.

## Conclusion

This analysis has identified important impacts of caring for a child with ADHD upon parent/carers hours of sleep and quality of sleep, satisfaction with leisure time, health (as measured by the EQ-5D though not the EQ-VAS or health satisfaction), life satisfaction, positive mental well-being, and happiness with relationships. The findings are not always robust to the inclusion of other variables, critically to employment status. However, it is not necessarily the case that the effects identified in the models which control for adult ADHD screen, employment, and relationship status are the most appropriate due to the potential inaccuracy of the ADHD screen and the potential for the caring role to have had a causal role in the current employment and relationship status. The substantial deficit experienced by the ADHD-group parents/carers in terms of sleep and leisure satisfaction suggests that these are potential areas in which greater support could be targeted. This could include consideration of more joint work between health and social care and the need for carer respite.

Identifying a clear pathway from caring for a child with ADHD to the health and quality of life impacts on the carers is difficult. This analysis clearly shows a negative impact across a range of health and broader quality of life outcomes, yet also reveals the complexity of isolating this impact from that of parents own ADHD, and shows the complex relationships among potential controls (employment, relationship status, income), health and quality of life outcomes, and family members ADHD status.

## Supplemental Material

Supplementary_On – Supplemental material for What Is the Health and Well-Being Burden for Parents Living With a Child With ADHD in the United Kingdom? Click here for additional data file. Supplemental material, Supplementary_On for What Is the Health and Well-Being Burden for Parents Living With a Child With ADHD in the United Kingdom? by Tessa Peasgood, Anupam Bhardwaj, John E. Brazier, Katie Biggs, David Coghill, David Daley, Cindy L. Cooper, Cyril De Silva, Val Harpin, Paul Hodgkins, Amulya Nadkarni, Juliana Setyawan and Edmund J. S. Sonuga-Barke in Journal of Attention Disorders

## Notes

1. The primary carers were identified as those who are responsible for a child between the ages of 0 and 18 years. The secondary cares were identified as those who are a parent or step-parent of a child aged 5 to 15 years, or they were more than 55 years. This was to exclude possible controls who, while living with a child, were not involved in the care of that child, for example, older siblings. The addition of the more than 55 years option was to include those who may be grandparents in the household.

2.
http://clahrc-sy.nihr.ac.uk/south-yorkshire-cohort.html

3. Up to two missing items can be tolerated (Kessler et al., 2006).
