# Supplementary material for: What Is the Health and Well-Being Burden for Parents Living With a Child With ADHD in the United Kingdom?
Source: J Atten Disord. 2020 Jun 19;25(14):1962–76. doi: 10.1177/1087054720925899 (PMC8527548; doi:10.1177/1087054720925899)
Supplement: Supplementary_On – Supplemental material for What Is the Health and Well-Being Burden for Parents Living With a Child With ADHD in the United Kingdom? [file Supplementary_On.pdf]

Supplementary On-line Material

Table S.1. Description of outcome measures (prior to matching and weighting)

Outcome Measures	ADHD-family group N=604 (unless otherwise specified)	South Yorkshire Cohort (SYC) N=214 (unless otherwise specified)	Understanding Society (USoc) N=8,595 (unless otherwise specified)
**Health and well-being**			
EQ-5D (0-1)	0.80 (sd .27)<br>range -.18 to 1<br>N=599	0.92 (sd .12)<br>range .29 to 1	NA
EQ-VAS (0-100)	74.30 (sd 20.17)<br>range 10 to 100<br>N=589	83.25 (sd 15.85)<br>range 10 to 100<br>N=210	NA
S-WEMWBS (7-35)<br>Positive mental health	20.94 (sd 3.80)<br>range 7 to 35<br>N=591	23.62 (sd 4.31)<br>range 7 to 35<br>N=210	22.46 (sd 3.94)<br>range 7 to 35<br>N=8273
Life satisfaction (1-7)	4.69 (sd 1.47)<br>range 1 to 7<br>N=600	5.48 (sd 1.18)<br>range (2 to 7)	5.05 (sd 1.47)<br>range 1 to 7<br>N=8,450
Health satisfaction (1-7)	4.64 (sd 1.62)<br>range 1 to 7<br>N=601	5.32 (sd 1.51)<br>range 1 to 7	4.92 (sd 1.65)<br>range 1 to 7<br>N=8,450
Leisure satisfaction (1-7)	3.59 (sd 1.67)<br>range 1 to 7<br>N=601	4.69 (sd 1.59)<br>range 1 to 7	4.24 (sd 1.57)<br>range 1 to 7<br>N=8,448
Income satisfaction (1-7)	4.19 (sd 1.65)<br>range 1 to 7<br>N=601	5.14 (sd 1.52)<br>range 1 to 7	4.24 (sd 1.71)<br>range 1 to7<br>N=8,440
Relationship happiness (1-7)<br>(those with a partner at home)	4.58 (sd 1.38)<br>range 1 to 7<br>N=462	4.99 (sd 1.10)<br>range 2 to 7<br>N=194	4.88(sd 1.33)<br>range 1 to 7<br>N=6,760
**Sleep**			
Hours of sleep	6.26 (sd 1.40)<br>range 1.33 to 14<br>N=573	7.05 (sd .93)<br>range 4 to 10.5<br>N=208	6.98 (sd 1.29)<br>range 0 to 16<br>N=8,141
Quality of sleep<br>• Very good<br>• Fairly good<br>• Fairly bad<br>• Very bad	 51 (8.6%)<br>265 (44.7%)<br>223 (37.6%)<br>54 (9.1%)<br>N=593	 55 (25.7%)<br>109 (50.9%)<br>45 (21.0%)<br>5 (2.3%)<br>N=214	 1,975 (23.0%)<br>4,461 (52.0%)<br>1,696 (19.8%)<br>444 (5.2%)<br>N=8,576
Trouble getting to sleep<br>• Not during the past month<br>• Less than once a week<br>• Once or twice a week<br>• Three or more times/ week<br>• More than once most nights	 35 (24.5%)<br>77 (14.0%)<br>78 (14.1%)<br>120 (21.7%)<br>142 (25.7%)<br>N=552	 94 (46.5%)<br>56 (27.7%)<br>26 (12.9%)<br>15 (7.4%)<br>11 (5.5%)<br>N=202	 3,462 (42.9%)<br>1,728 (21.4%)<br>1,239 (15.3%)<br>790 (9.8%)<br>855 (10.6%)<br>N= 8,074
Waking up too early<br>• Not during the past month<br>• Less than once a week	 57 (10.0%)<br>50 (8.8%)	 29 (13.7%)<br>50 (23.7%)	 1,971 (24.1%)<br>1,418 (17.3%)

• Once or twice a week<br>• Three or more times/ week<br>• More than once most nights	94 (16.5%)<br>143 (25.1%)<br>266 (39.7%)<br>n=570	43 (20.4%)<br>48 (22.8%)<br>41 (19.4%)<br>n=211	1,740 (21.3%)<br>1,530 (18.7%)<br>1,519 (18.6%)<br>n=8,178
Number of times carer is woken by the most unsettled child (main carer only)	None = 155<br>1 time = 51<br>2 times =61<br>3 times =13<br>4 times =6<br>5 to 9 times = 13	None = 87<br>1 time = 7<br>2 times =2<br>3 or more times =0	NA

Note: we do not show tests of significant difference between these outcomes since they are all compared in the data analysis using methods which control for important differences in background characteristics between the groups.

Table S.2 Marginal effects of reporting at least ‘some’ problems in the individual items of the EQ-5D. Based on a logit model, using the SYC controls; matched and weighted. Controlling for standard controls and adult ADHD screen.

EQ-5D items	Marginal effect of having a child with ADHD
Mobility : “I have some problems in walking about / I am confined to bed”	-.0001
Self-care: “I have some problems washing or dressing myself / I am unable to wash or dress myself”	.0484***
Usual activities: “I have some problems with performing my usual activities / I am unable to perform my usual activities”	-.0471
Pain/discomfort: “I have moderate pain or discomfort / I have extreme pain or discomfort”	-.0414
Depression/anxiety: “I am moderately anxious or depressed / I am extremely anxious or depressed	.1707***
N	687

*Notes: Controls include: age, gender, number of children in the household, % employment deprived in the area and % income deprived, highest education level attained, secondary carer and adult ADHD screen. A constant is also included. *** p<0.01, ** p<0.05, * p<0.1, these are based on robust standard errors which are clustered at the household level. Full details of these regressions are available from the authors. One respondent reported level 3 in self- care, however, the main findings were robust to their exclusion.*

Table S.3 Marginal effects of having a child with ADHD on the probability of reporting ‘often’ or ‘all the time’ to items in the S-WEMWBS. Based on a logit model; matched and weighted.

Reporting often or all of the time. S-WEMWB items	USoc Controlling for standard controls.	SYC Controlling for standard controls and adult ADHD screen.
Optimism: “I’ve been feeling optimistic about the future”	-.4692 ***	-.2450 ***
Useful: “I’ve been feeling useful”	-.1392 ***	-.0728
Relaxed: “I’ve been feeling relaxed”	-.2041 ***	-.2138 ***
Dealing with problems: “I’ve been dealing with problems well”	-.1453 ***	-.1896 ***
Thinking clearly: “I’ve been thinking clearly”	-.1628 ***	-.1100 *
Close to others: “I’ve been feeling close to other people”	-.1202 ***	-.0669
Make up mind: “I’ve been able to make up my own mind about things”	-.1094 ***	-.0701
N	9006	689

Notes: Controls include: age, gender, number of children in the household, % employment deprived in the area and % income deprived, highest education level attained, secondary carer and for the SYC controls only, adult ADHD screen. A constant is also included. *** $p<0.01$, ** $p<0.05$, * $p<0.1$, these are based on robust standard errors which are clustered at the household level. Full details of these regressions are available from the authors.
